# Exploring associations between nasal fluid inflammatory proteins and patient-reported symptom burden in respiratory disease

**DOI:** 10.3389/falgy.2026.1902168

**Published:** 2026-07-29

**Authors:** Tanya Lupancu, Sharmala Thuraisingam, Eldin Rostom, David M. Yen, Brian S. Wang, Adam M. Damry

**Affiliations:** 1 Diag-Nose Medical, Notting Hill, VIC, Australia; 2 Department of Surgery, The University of Melbourne, Fitzroy, VIC, Australia; 3 Specialty Physician Associates, Bethlehem, PA, United States; 4 Department of Chemistry, University of Ottawa, Ottawa, ON, Canada

**Keywords:** airway inflammation, biomarkers, nasal fluid, respiratory disease, SNOT-22

## Abstract

**Introduction:**

Patient-reported outcome measures are widely used to assess symptom burden in respiratory disease, yet the extent to which symptom severity reflects underlying mucosal biology remains incompletely understood. Nasal fluid provides a non-invasive matrix for assessing airway inflammation within the unified airway framework.

**Methods:**

In this exploratory study, nasal fluid samples were collected from 30 participants with heterogeneous respiratory conditions, and protein concentrations were measured using a multiplex immunoassay. Associations between protein concentrations and symptom burden, measured using the 22-item Sino-Nasal Outcome Test (SNOT-22), were assessed using Spearman correlations.

**Results:**

Protein concentrations of 40 analytes were measured and were found to span a wide dynamic range across participants. The mean SNOT-22 score was 39.1 (range, 3-89). TRAIL and CXCL10 demonstrated positive correlations with SNOT-22 scores (rho = 0.563, *p* = 0.001 and rho = 0.520, *p* = 0.005, respectively). Heatmap analysis revealed marked inter-individual variability in nasal fluid protein expression, including among participants with similar SNOT-22 scores.

**Discussion:**

These findings demonstrate the feasibility of linking non-invasive nasal fluid biomarkers with composite symptom scores in a heterogeneous respiratory cohort. Together, they support the potential of nasal fluid profiling to capture clinically relevant biological variation and inform larger, multimodal studies integrating complementary clinical and physiological measures.

## Introduction

Respiratory diseases are characterized by substantial heterogeneity in both clinical presentation and inflammatory mechanisms, and overlapping symptoms are frequently observed across upper and lower airway conditions. Within the unified airway framework, the nasal and bronchial compartments are recognized as anatomically and immunologically connected, with shared inflammatory pathways contributing to disease activity across the respiratory tract (1, 2). However, clinical assessment and disease monitoring are still most often performed using disease-specific tools and care pathways, which may not fully capture overlapping or coexisting inflammatory processes across airway compartments.

Patient-reported outcome measures play an important role in assessing symptom burden and disease impact in respiratory medicine. However, many commonly reported symptoms, such as nasal obstruction, discharge, cough, sleep disturbance, and fatigue, are not specific to a single diagnosis and may reflect different underlying biological mechanisms depending on the disease context and patient phenotype (3–5). In addition, disease activity is often evaluated using a combination of questionnaires, physiological tests, imaging, and biomarkers, which vary across conditions and clinical settings (6–8). As a result, assessment approaches can be difficult to compare across respiratory diseases and may not consistently clarify relationships between patient-reported symptoms and underlying inflammatory processes.

The 22-item Sino-Nasal Outcome Test (SNOT-22) is a validated patient-reported outcome measure used to assess sinonasal symptom burden and health-related quality of life across rhinologic, functional, sleep, and emotional domains. Originally developed and validated for chronic rhinosinusitis, it has also been applied in CRS populations that include patients with comorbid asthma (9, 10). As a composite instrument, SNOT-22 captures overall symptom burden rather than specific underlying biological pathways. Accordingly, reported associations between SNOT-22 scores and individual inflammatory markers have been variable and appear to depend on factors such as disease phenotype, sample matrix and collection site, and analytical approach (11–13).

Previous work has shown that inflammatory mediators measured in nasal mucus, lavage, or tissue can be associated with symptom burden and disease severity in selected airway disease cohorts (14–16); however, reported relationships have been variable across studies and often depend on factors such as disease phenotype, sampling site and method, and analytical approach (17, 18). Moreover, most studies have examined single diagnostic groups, meaning patients with co-existing respiratory conditions are often under-represented, despite this being common in clinical practice (19–22). As a result, it remains less clear how mucosal inflammatory profiles relate to composite symptom scores in mixed respiratory populations with overlapping symptoms and inflammatory pathways.

Nasal fluid represents a practical sample matrix for studying airway inflammation within the unified airway framework. Because it is derived from the nasal mucosal environment, nasal fluid can provide information on local immune and inflammatory activity at the interface between environmental exposure and host response, while remaining amenable to non-invasive and repeatable sampling (18, 23–25). Advances in multiplex proteomic technologies now enable simultaneous quantification of a broad range of inflammatory mediators from small nasal fluid volumes, supporting exploratory analyses of inflammatory heterogeneity across respiratory conditions using a common biological readout (26, 27).

The primary objective of this exploratory study was to assess whether protein biomarkers measured in nasal fluid demonstrate measurable associations with patient-reported symptom burden, using SNOT-22 as an initial clinical outcome measure in a mixed respiratory cohort. By examining a broad panel of inflammatory proteins and relating their concentrations to a widely used composite symptom score, this study aimed to evaluate the feasibility of linking nasal fluid biology with patient-reported outcomes across overlapping respiratory conditions and to identify candidate biomarkers for further investigation in larger, more phenotypically resolved studies.

## Materials and method

### Study design and inclusion/exclusion criteria

The study protocol was approved and monitored by the Advarra Institutional Review Board and conducted in accordance with the principles of the Declaration of Helsinki. Written informed consent was obtained from all participants prior to any study-related procedures.

Eligible participants were adults aged 18 years or older with moderate rhinorrhea who were able to produce at least 0.5 mL of nasal fluid by nose blowing. Participants were excluded if they were unable to blow their nose or could not collect the required volume of nasal secretions within a 5-minute period, as determined by the investigator.

Participants were recruited from a respiratory research cohort and included individuals with respiratory conditions identified by the investigator based on clinical evaluation and standard diagnostic frameworks, including asthma, chronic rhinosinusitis, rhinitis, type 2 inflammation, and respiratory infection. Diagnostic categories were not used for stratification or statistical analysis.

### Sample and data collection

Blown nasal secretions were collected from participants with moderate to severe rhinorrhea. Nasal secretions were collected as blown nasal fluid by an ENT specialist. Participants blew nasal secretions into a sterile specimen collector over a period of 5 min or less, to collect approximately 0.5 cc, which were then stored at −80 °C until analysis. Participants additionally completed a comprehensive medical history questionnaire and the 22-item Sino-Nasal Outcome Test (SNOT-22) to assess symptom severity. The SNOT-22 consists of 22 items scored from 0 (no problem) to 5 (problem as bad as it can be), with total scores ranging from 0 to 110; higher scores indicate greater symptom burden and worse disease-specific quality of life. Relevant demographic and clinical characteristics are summarized in Table 1.

**Table 1:** Patient demographics and clinical characteristics.

Demographic Factor	Category	*n*	%
Recruited patients		30	100%
Age, years	Median 37.5 (range 18–74)		
Sex	Female	18	60%
	Male	12	40%
Ethnicity	Black or African American	3	10%
	Asian	3	10%
	Hispanic or Latino	1	3%
	White	16	53%
	Multi-racial	6	20%
Asthma Status	Yes	7	23%
	No	23	77%
Smoking Status	Current Smoker	7	23%
	Non-Smoker (never or former)	23	77%
Prior Nasal Surgery	Yes	3	10%
	No	27	90%
Medication Usage^a^	Yes	11	37%
	No	19	63%

a Medication use was self-reported at the time of sample collection.

### Protein analysis

Nasal secretion samples were analyzed using a 40-plex immunoassay performed by Crux Labs (Australia). Samples were diluted 1:50 in the assay diluent supplied with the kit, and 50 µL of the diluted sample was loaded per well, in accordance with the manufacturer's instructions and following common clinical sampling and assay procedures. Assays were run on the MAGPIX system using xPONENT software according to the manufacturer's instructions (Thermo Fisher Scientific). Data processing and quantification were conducted using Belysa Analysis Software (MilliporeSigma).

The analyte panel included inflammatory cytokines, chemokines, proteases, and regulatory mediators, each with defined lower limits of detection (LLOD). These included C-Reactive protein (CRP; LLOD: 7 pg/mL), C-X-C Motif Chemokine Ligand 10 (CXCL10/ IP-10; LLOD: 2 pg/mL), Epidermal Growth Factor (EGF; LLOD: 2.5 pg/mL), Eotaxin-1 (also known as CCL11; LLOD: 0.6 pg/mL), Eotaxin-2 (CCL24; LLOD: 2.2 pg/mL), Eotaxin-3 (CCL26; LLOD: 0.2 pg/mL), Fractalkine (CX3CL1; LLOD: 1.1 pg/mL), granulocyte-colony stimulating factor (G-CSF; LLOD: 14.5 pg/mL), granulocyte macrophage-colony stimulating factor (GM-CSF; LLOD: 20.6 pg/mL), GzmA (Granzyme-A; LLOD: 8.4 pg/mL) and GzmB (Granzyme-B; LLOD 8.1 pg/mL), interferon (IFN) -alpha (LLOD: 0.6 pg/mL), IFN*γ* (LLOD: 9.4 pg/mL), interleukin 1 receptor antagonist (IL-1RA; LLOD: 40 pg/mL), interleukin (IL) −2 (LLOD: 9.8 pg/mL), IL-3 (LLOD: 20.5 pg/mL), IL-4 (LLOD: 15.1 pg/mL), IL-5 (LLOD:10 pg/mL), IL-6 (LLOD: 12.8pg/mL), IL-8 (LLOD: 2.5 pg/mL), IL-9 (LLOD: 8.2 pg/mL), IL-10 (LLOD: 1.5 pg/mL), IL-12p70 (LLOD: 7.9 pg/mL), IL-13 (LLOD: 4.1 pg/mL), IL-22 (LLOD: 24.2 pg/mL), IL-37 (LLOD: 3.4 pg/mL), monocyte chemoattractant protein-1 (MCP-1, also known as CCL2; LLOD: 4.2 pg/mL), macrophage inflammatory protein −1 (MIP-1) -*α* (LLOD: 18.5 pg/mL), MIP-1β (LLOD: 8 pg/mL), matrix metalloproteinases (MMP) −1 (LLOD: 7 pg/mL), MMP-7 (LLOD: 5.1 pg/mL), MMP-8 (LLOD: 24.8 pg/mL), myeloperoxidase (MPO; LLOD: 76.6 pg/mL), serum amyloid A (SAA; LLOD: 42.4 pg/mL), tumor necrosis factor (TNF) -*α* (LLOD: 5.6 pg/mL), TNF*β* (LLOD: 6.1 pg/mL), TNF-Related Apoptosis Inducing Ligand (TRAIL; LLOD: 2 pg/mL) and Triggering Receptor Expressed on Myeloid Cells (TREM) −1 (LLOD: 24.7 pg/mL) and TREM-2 (LLOD: 24.7 pg/mL).

### Data management and statistical analysis

All identifying information was removed prior to analysis, and anonymized data were securely stored in an encrypted, HIPAA-compliant REDCap database (Vanderbilt University). Only analytes quantified above the LOQ in at least 80% of samples were retained for downstream analyses, excluding those with higher missingness to limit the influence of non-detect values on the correlation analyses. Concentrations below the analyte-specific LLOD were imputed as one-tenth of the corresponding LLOD. Biomarker concentrations were log10-transformed prior to statistical analysis.

Due to the modest sample size and limited medication detail, analyses were not adjusted for medication use. Statistical analyses were performed using GraphPad Prism version 10. Associations between nasal fluid protein concentrations and SNOT-22 scores were assessed using nonparametric Spearman correlation. Statistical significance was defined as *p* < 0.05, with significance levels denoted as *p < 0.05, **p < 0.01 and ***p < 0.001.

## Results

### Participant characteristics

A total of 30 participants were included in the analysis. The median age was 37.5 years (range, 18–74), and 60% of participants were female (Table 1). The cohort was racially diverse, with 53% identifying as white, 20% as multiracial, 10% as Black or African American, 10% as Asian, and 3% as Hispanic or Latino. 30% had allergic rhinitis, 13% had non-allergic rhinitis, 33% had a viral respiratory infection, 3% had chronic rhinosinusitis, and 20% had no reported respiratory diagnosis.

Current smoking was reported by 23% of participants, while the remaining 77% were non-smokers. Three participants (10%) reported a history of prior nasal surgery. Medication use at the time of sample collection was reported by 37% of participants. Overall, this cohort represented a heterogeneous population suitable for exploratory biomarker analyses. Given this heterogeneity, this study was designed to evaluate feasibility and identify candidate symptom-associated biomarkers rather than establish disease-specific inflammatory signatures.

### Correlation of SNOT-22 scores and nasal fluid biomarker concentrations

**Figure 1:**
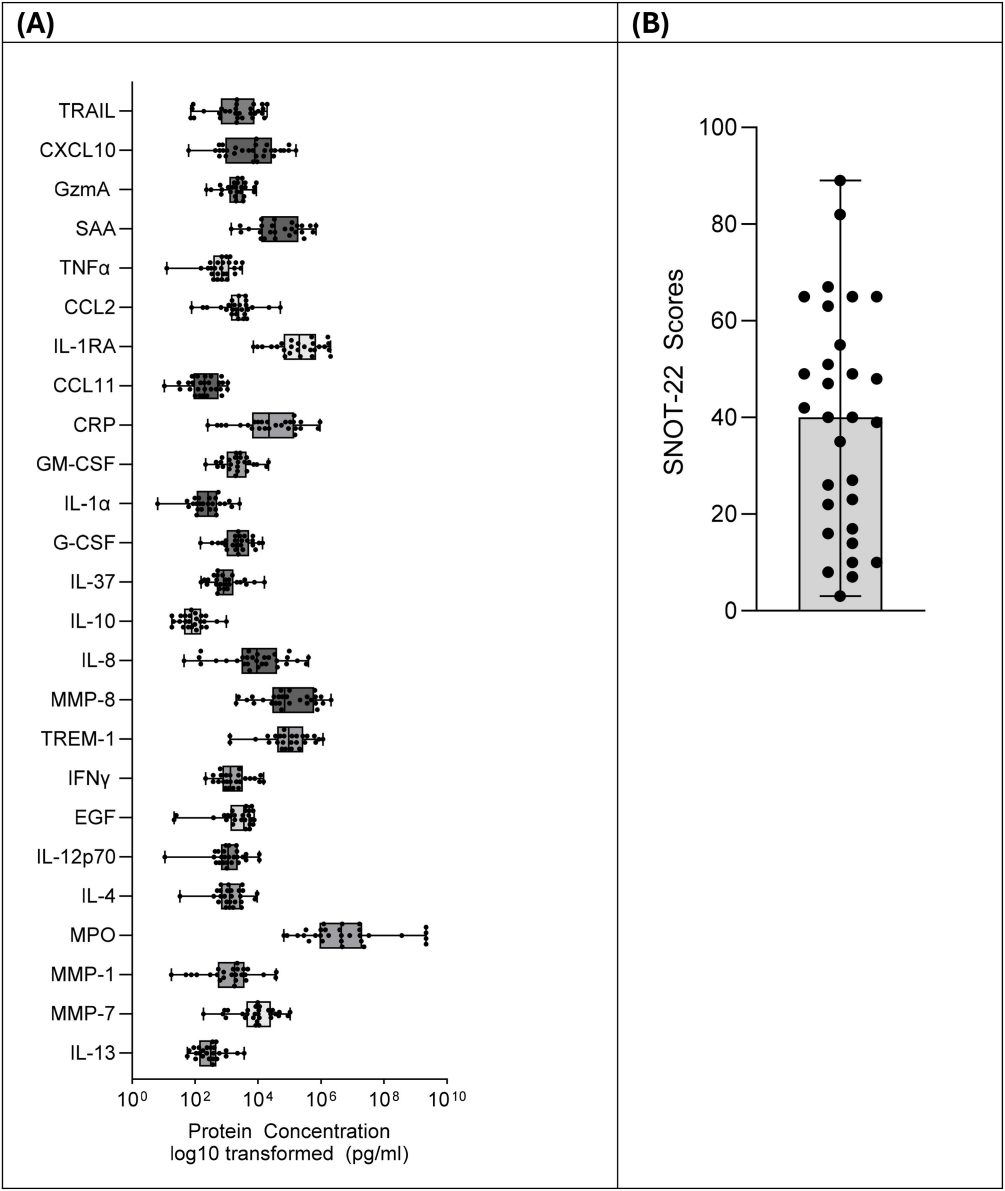
Distribution of nasal fluid biomarker protein concentrations and SNOT-22 scores. **(A)** Protein biomarkers detected in over 80% of nasal fluid samples from 30 participants are shown as box-and-whisker plots. Boxes represent the interquartile range (25th–75th percentiles), the central line indicates the median, and whiskers denote the minimum to maximum values; individual data points are overlaid. **(B)** SNOT-22 scores are shown as individual participant values overlaid on bars representing the median, with bars extending from the minimum to the maximum observed scores.

Of the 40 analytes measured in nasal fluid samples, 25 met the predefined data completeness criterion and were included in subsequent analyses (Figure 1A). These analytes include CCL2, CCL11, CRP, CXCL10, EGF, G-CSF, GM-CSF, GzmA, IFNγ, IL-1RA, IL-1a, IL-4, IL-8, IL-10, IL12p70, IL-13, IL-37, MMP1, MMP7, MMP8, MPO, SAA, TNF*α*, TRAIL, TREM-1. CCL24, CCL26, CX3CL1, GzmB, IFN*α*, IL-2, IL-3, IL-5, IL-6, IL-9, IL-22, MCP-1, MIP-1*α*, TNF*β*, TREM-2, were excluded due to a high proportion of values below the lower limit of detection. Among the included analytes, protein concentrations generally spanned approximately 10^2^ to 10^6^ pg/mL. MPO exhibited the widest concentration range, extending from approximately 10^5^ to 10^10^ pg/mL.

The mean SNOT-22 score was 39.1, with values ranging from 3 to 89 (Figure 1B).

**Figure 2:**
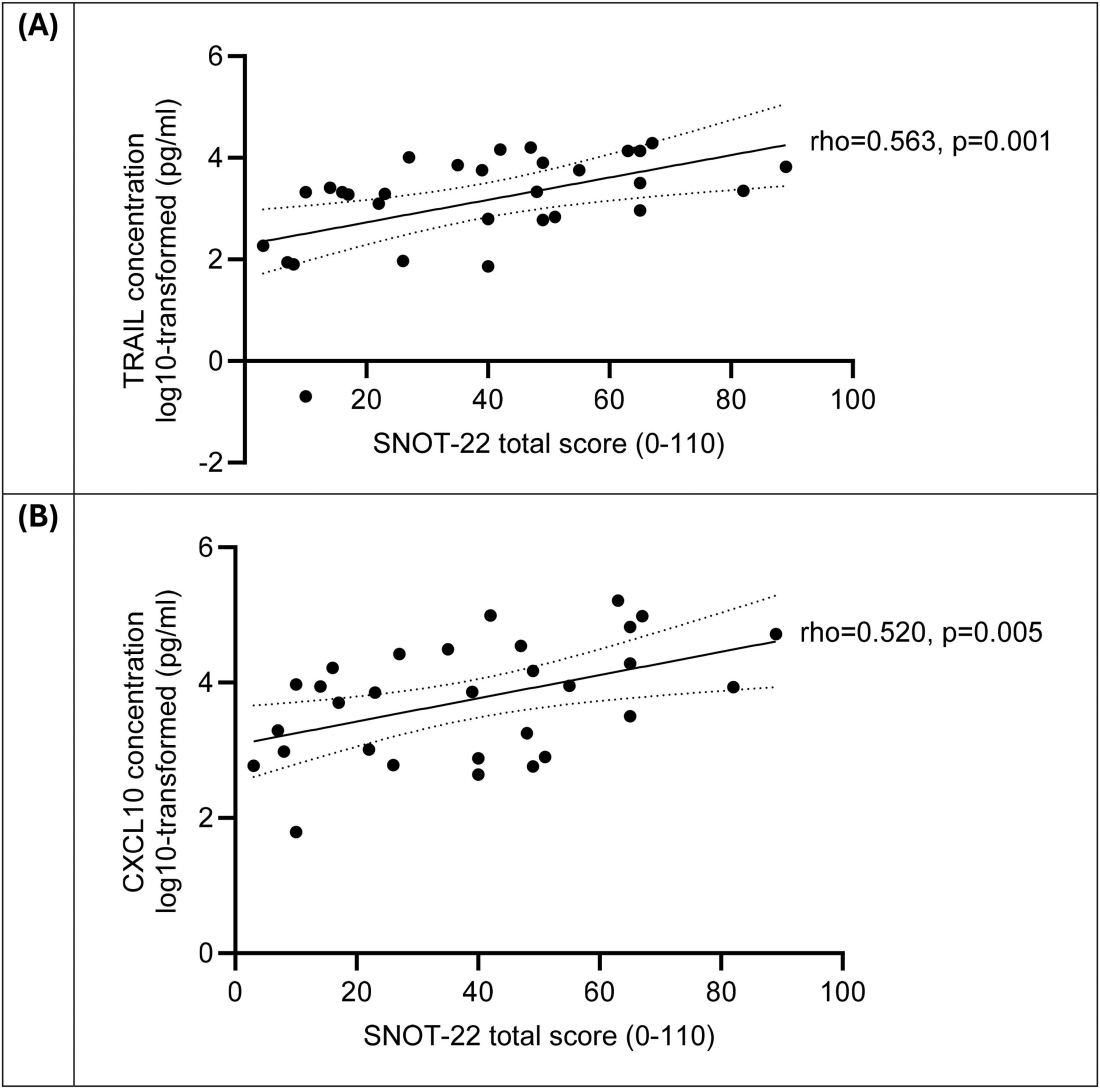
Association between intranasal biomarker concentration and symptom burden. Scatter plots show Spearman correlations between SNOT-22 total score and **(A)** TRAIL and **(B)** CXCL10 protein concentrations. Each point represents an individual participant (*n* = 30), ordered by increasing SNOT-22 total score to visualise changes in intranasal inflammatory profiles across symptom burden. A linear model was fitted to the data for visualisation only; dotted lines represent 95% confidence bands; rho represents the Spearman rank correlation coefficient.

Spearman correlation analyses for all included proteins are summarized in Table 2. TRAIL (*ρ* = 0.563, *p* = 0.001, q = 0.037) and CXCL10 (*ρ* = 0.520, *p* = 0.005, q = 0.058) demonstrated statistically significant positive correlations with SNOT-22 scores, as shown in Figure 2. No other protein concentrations showed statistically significant associations with SNOT-22 scores.

**Table 2:** Correlation between SNOT-22 scores and cytokine concentrations.

Biomarker	*n*	Spearman rho (95% CI)	*p* (unadjusted)	q (BH-adjusted)	FDR < 0.05
TRAIL	29	0.563 (0.220 to 0.782)	0.001	0.037	*
CXCL10	28	0.520 (0.157 to 0.759)	0.005	0.058	
GzmA	25	0.328 (−0.088 to 0.647)	0.109	0.839	
SAA	27	0.271 (−0.129 to 0.595)	0.172	0.839	
TNFα	26	0.259 (−0.149 to 0.592)	0.201	0.839	
CCL2	24	−0.253 (−0.600 to 0.174)	0.233	0.839	
IL-1RA	29	0.207 (−0.177 to 0.536)	0.282	0.839	
CCL11	24	0.209 (−0.217 to 0.568)	0.327	0.839	
CRP	28	0.187 (−0.203 to 0.526)	0.34	0.839	
GM-CSF	25	−0.186 (−0.544 to 0.229)	0.372	0.839	
IL-1α	25	0.179 (−0.236 to 0.538)	0.393	0.839	
G-CSF	24	−0.177 (−0.544 to 0.247)	0.409	0.839	
IL-37	25	−0.157 (−0.522 to 0.256)	0.453	0.839	
IL-10	24	−0.154 (−0.526 to 0.269)	0.473	0.839	
IL-8	24	−0.144 (−0.519 to 0.278)	0.503	0.839	
MMP-8	27	0.101 (−0.291 to 0.464)	0.617	0.897	
TREM-1	24	−0.108 (−0.491 to 0.310)	0.617	0.897	
IFNγ	25	0.097 (−0.311 to 0.474)	0.646	0.897	
EGF	25	−0.078 (−0.459 to 0.328)	0.712	0.899	
IL-12p70	29	0.070 (−0.305 to 0.426)	0.719	0.899	
IL-4	25	−0.059 (−0.444 to 0.345)	0.781	0.93	
MPO	25	0.034 (−0.366 to 0.424)	0.871	0.962	
MMP-1	25	0.030 (−0.369 to 0.421)	0.885	0.962	
MMP-7	24	−0.020 (−0.420 to 0.387)	0.926	0.965	
IL-13	25	0.005 (−0.391 to 0.399)	0.981	0.981	

BH, Benjamin-Hochberg; FDR, false-discovery rate adjustment.

### Nasal fluid protein expression patterns across SNOT-22 severity

**Figure 3:**
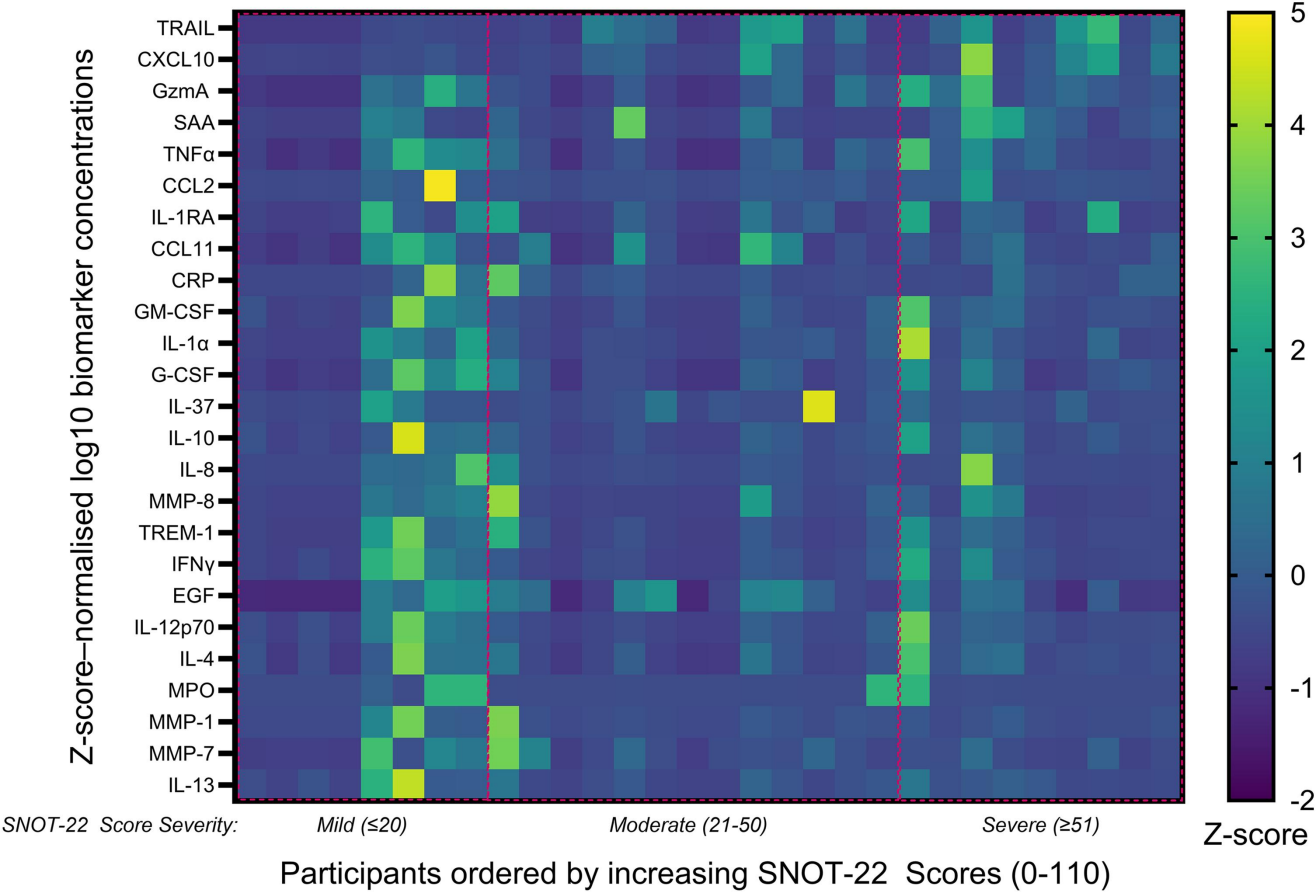
Exploratory heatmap of intranasal inflammatory biomarker profiles ordered by symptom burden. Heatmap shows z-score–normalised log10-transformed concentrations of 25 intranasal inflammatory biomarkers across 30 participants. Samples are ordered by strength of correlation between cytokine concentrations and SNOT-22 total score. Red dashed boxes group SNOT-22 scores into severity categories as mild (≤20), moderate (21-50), and severe (≥51). This analysis is exploratory and hypothesis-generating.

Z-score–normalized log10-transformed nasal fluid protein concentrations are shown as a heatmap with participants ordered by increasing SNOT-22 scores (Figure 3). Substantial inter-individual variability was observed across most analytes, with no consistent pattern of coordinated expression across proteins. Visual inspection of the heatmap suggested some increases in protein expression, particularly among participants with SNOT-22 scores ranging from 10 to 23; however, this pattern is driven by a small number of participants with markedly elevated concentrations across multiple analytes. Overall, protein expression patterns were heterogeneous across the SNOT-22 spectrum, with no clear monotonic relationship to symptom severity, including among participants with similar SNOT-22 scores. This aligns with the preceding correlation analysis, in which only two proteins showed moderate correlations with SNOT-22 scores.

The substantial variability observed in inflammatory protein expression among participants with comparable symptom scores indicates heterogeneity in inflammatory profiles across the cohort rather than a uniform biological phenotype. Such heterogeneity is well recognized in airway disease, where overlapping type 1, type 2, and innate immune pathways can contribute to similar clinical presentations (28–31). In a mixed respiratory cohort with variable asthma status, smoking exposure, and medication use, this diversity is expected and underscores the limitations of symptom-based measures alone in fully characterizing inflammatory biology.

## Discussion

The primary aim of this exploratory study was to evaluate whether nasal fluid protein biomarkers demonstrate measurable associations with patient-reported symptom burden, using SNOT-22 as an initial clinical outcome measure in a mixed respiratory cohort. Within this framework, CXCL10 and TRAIL were identified to show positive correlations with SNOT-22 scores. Although these associations were moderate in magnitude, they support the broader premise that nasal fluid biology can reflect aspects of patient-reported symptom severity, extending prior observations by demonstrating this relationship in a mixed respiratory cohort using a non-invasive nasal fluid matrix.

Interpretation of these findings should consider the composite nature of SNOT-22, which captures sinonasal, sleep, and psychosocial symptom domains rather than activity within a single inflammatory pathway (32, 33). In a heterogeneous cohort containing allergic, infectious, and chronic inflammatory airway conditions, it is therefore unsurprising that only a subset of biomarkers demonstrated monotonic relationships with total symptom burden. Some biomarkers may relate more strongly to specific symptom domains or objective physiological measures than to aggregate SNOT-22 score. In this context, associations with CXCL10 and TRAIL are consistent with prior airway disease literature and support further investigation of these pathways in relation to patient-reported symptom burden.

CXCL10 (IP-10) is an interferon-inducible chemokine that promotes recruitment of activated T cells and other immune effectors to inflamed tissue (34, 35). Prior studies have reported elevated CXCL10 expression in allergic rhinitis, chronic rhinosinusitis, and virus-associated airway inflammation (36). Taken together, these findings support the interpretation that elevated CXCL10 in nasal fluid can reflect heightened interferon-linked immune activation and immune-cell recruitment, processes that plausibly contribute to congestion, discharge, facial pressure, and sleep impairment; symptom domains incorporated within the SNOT-22 score.

TRAIL (TNFSF10) is a TNF superfamily ligand that mediates both apoptotic and non-apoptotic inflammatory signalling and is linked to NF-*κ*B- and MAPK-associated pathways that contribute to immune activation and epithelial–immune interactions (37). Elevated sputum TRAIL has been associated with greater inflammatory burden, including increased airway leukocytes and cytokine activity (38). Although mechanistic conclusions cannot be drawn from this study, the observed relationship between TRAIL and SNOT-22 supports further investigation of TRAIL-associated inflammatory pathways in heterogeneous airway disease populations.

The modest strength of the observed correlations, together with the absence of significant associations for most analytes, highlights the complexity of symptom–biology relationships in airway disease. These observations suggest that a single composite symptom score cannot capture biologically diverse inflammatory processes across individuals (28, 39). However, the absence of associations for other cytokines commonly implicated in airway inflammation, including type 2- and neutrophil-associated markers, does not preclude their relevance but may reflect cohort heterogeneity, treatment effects, limited sample size, and the cross-sectional design (28, 29). Similar variability has also been reported in prior studies examining nasal mucus and lavage biomarkers across chronic rhinosinusitis, allergic rhinitis, and asthma cohorts, where associations often depend on disease phenotype, inflammatory endotype, and outcome measures used (14, 16, 40–42).

Despite limited single-biomarker correlations with total SNOT-22 score, however, the broad detection of inflammatory mediators across nasal fluid samples supports the feasibility of multiplex nasal fluid profiling in respiratory biomarker research. Some biomarkers may ultimately prove more informative when interpreted alongside narrower symptom domains or objective physiological measures rather than composite symptom burden alone. Accordingly, a stratified analysis with a larger overall sample size may lead to the identification of additional correlative biomarkers that can identify disease in stratified subgroups. However, the modest overall sample size and per-subgroup numbers in this study preclude such an analysis due to statistical underpowering, and the reported correlations require validation in larger, adequately powered and more homogeneous cohorts before generalisation. Future studies integrating nasal fluid biomarkers with additional patient-reported outcomes that target narrower symptom constructs, as well as objective measures such as fractional exhaled nitric oxide (FeNO), spirometry, or other lung function metrics, may additionally improve the resolution of biologically meaningful patterns.

Several limitations should be acknowledged. Given the exploratory nature of the study, sample size was determined by the number of eligible participants recruited during the study period rather than a discrete calculation method. Although Benjamini–Hochberg false-discovery-rate adjustment was applied and q-values are reported, the number of comparisons and modest sample size increase the possibility that some observed associations represent false positives, warranting cautious interpretation. The cross-sectional design limits inference regarding temporal relationships between biomarkers and symptom burden, and the use of a single questionnaire restricts interpretability across different respiratory symptom constructs. Although the study deliberately sampled across a range of disease activity, inclusion of patients with viral infection may have further influenced both secretion volume and nasal protein levels in this subset of patients. Additionally, exclusion of analytes with a high proportion of values below the lower limit of detection may have limited assessment of low-abundance but biologically relevant proteins.

In summary, this exploratory study identified moderate positive associations between nasal fluid CXCL10 and TRAIL concentrations and patient-reported symptom burden in a heterogeneous respiratory cohort. The findings support the feasibility of linking non-invasive nasal fluid biomarker profiling with clinical symptom measures while also highlighting substantial inter-individual inflammatory heterogeneity and the limitations inherent in single-questionnaire approaches. Larger longitudinal studies with deeper clinical phenotyping and objective physiological assessment will be required to determine how nasal fluid biomarkers may complement symptom-based respiratory disease evaluation.

## Data Availability

The original contributions presented in the study are included in the article/Supplementary Material, further inquiries can be directed to the corresponding author.
